# Managing Anaemia in Pregnancy with Traditional Medicines: Experiences of women in the Cape Coast Metropolis, Ghana

**DOI:** 10.1371/journal.pone.0345114

**Published:** 2026-04-06

**Authors:** Theodora Dedo Azu, Margaret Nyarko-Sampson, Esther Danquah, Patience Fakornam Doe

**Affiliations:** 1 Department of Maternal and Child Health Nursing, University of Cape Coast, College of Health and Allied Sciences, School of Nursing and Midwifery, Cape Coast, Ghana; 2 Faculty of Health Sciences, North West University, Centre for Health Professions Education, Potchefstroom, South Africa; 3 Ghana Health Service, Central Regional Health Directorate, Cape Coast, Central Region, Ghana; 4 Nottingham University Hospital NHS Trust – City Campus: Nottingham City Hospital. Queen’s Medical Centre, Nottingham, United Kingdom; 5 Department of Public Health Nursing, University of Cape Coast, School of Nursing and Midwifery, Cape Coast, Ghana; Kwame Nkrumah University of Science and Technology Faculty of Pharmacy and Pharmaceutical Sciences, GHANA

## Abstract

Anaemia in pregnancy (AIP) is diagnosed when a pregnant woman’s haemoglobin levels fall below 11g/dL. Globally, traditional medicines (TM) are widely used in the management of pregnancy-related conditions. This study aims to explore the experiences and management practices associated with the use of TMs to manage AIP.

**Materials and methods:**

A qualitative exploratory descriptive design was used to explore the experiences of pregnant women. Semi-structured face-to-face interviews were conducted among 18 pregnant women during their antenatal visits. Interviews were audio-recorded and transcribed verbatim for analysis. The researchers developed a codebook and used NVIVO 12 to analyse the transcribed data,

**Results and analysis:**

The women and their partners had lower socio-economic status (education, occupation and monthly income). The women selected for the study knew about the causes of AIP, including poor medication adherence and their inability to eat well. The women used TMs to manage AIP; however, they were concerned about the side effects on themselves and their fetuses. Although health workers discouraged the women from using TMs, family, friends, and communities encouraged their use.

**Conclusion:**

The women in this study perceived that traditional medicines could help manage AIPs, although they also reported concerns about adverse effects following use. Given the widespread accessibility of these remedies, further research is needed to clarify their safety, potential benefits, and appropriate use. Rather than outright discouragement, efforts should focus on strengthening supervised care and referral pathways.

## Introduction

Anaemia is defined as a low haemoglobin concentration in the blood [1]. Anaemia in pregnancy (AIP) is diagnosed when a pregnant woman’s haemoglobin level falls below 11g/dL [2]. Anaemia in women is particularly heightened during pregnancy and postnatal periods due to higher nutritional demands and a deficient iron supply [3]. Anaemia-related risks associated with morbidity and mortality in pregnancy are evidenced in mothers, and their effects are later visible in the neonate [4].

Interventions to prevent AIP-related risk factors include family planning, promoting the utilization of insecticide-treated nets, intermittent preventive therapy for malaria, and iron and folic acid supplementation [5]. Although effective management of AIP includes iron and folic acid supplementation, some women do not comply with medication regimens or prefer Traditional Medicines (TMs). Several TMs have been perceived to be highly effective in managing maternal health symptoms in pregnancy, including tiredness, pallor, fatigue, etc.

Traditional medicines are used in various parts of the world, especially in Africa, to manage maternal health issues [6]. In sub-Saharan Africa, women often use TMs due to limited access to healthcare services [7,8], and their perception of enhancing the health and intelligence of their fetuses [9]. Current evidence on how often TMs are prescribed and used during pregnancy remains limited, especially in Africa, because of poor documentation of specific traditional dosages. These knowledge gaps make TM use in pregnancy, especially for the management of AIP, a public health concern, as the effects and safety of many such remedies during pregnancy are not well understood.

There is a paucity of literature on the utilisation of TMs in the management of AIP in Ghana. This study aims to explore women’s experiences of Anaemia in Pregnancy and their management practices for the condition using Traditional Medicines in the Cape Coast Metropolis.

## Methods and methodology

### Study design

Study design refers to a general plan for implementing a research strategy [10]. A qualitative, exploratory, and descriptive design was adopted to explore women’s perceptions of the management of AIP with TMs. The study sought to generate an in-depth, context-specific understanding of women’s perceptions and lived experiences of managing AIP with TMs, an area where local empirical evidence is limited and key details, such as TM types, preparation, and dosing, are poorly documented.

### Study settings

Cape Coast Teaching Hospital and the Cape Coast Metropolitan Hospitals’ antenatal clinics were purposively selected for the study. This was because they are high-volume referral, primary and secondary care facilities, respectively, that provide antenatal services to women from diverse backgrounds within Cape Coast Metropolis and its environs.

### Sampling approach for pregnant women

A non-probability sampling approach was used to select pregnant women for this study. Semi-structured face-to-face interviews were conducted among 18 pregnant women, nine from each study setting. The midwives helped the researcher to identify potential participants as they arrived in the morning for routine Antenatal care (ANC). The researcher then approached the women and explained the study. Pregnant women who had at least one child were included in the study, and those who were clinically unwell were excluded from the study. Eligibility was determined prior to inviting them to participate in the study.

### Data collection

This phase explored pregnant women’s perceptions of the integration of TMs in the management of AIP, using semi-structured, face-to-face individual interviews that lasted approximately 40 minutes from the 1^st^ February, 2024–30^th^ June, 2024. The interviews were conducted by the principal investigator in a designated quiet room within the selected hospitals. Written informed consents were obtained from the participants before the interviews.

All participants were informed that the interviews would be audio-recorded for transcription purposes. The researchers initiated and sustained a critical dialogue, listening analytically throughout the interview period [11], which helped maintain a cordial atmosphere during the data collection phase. Data saturation was reached after 9 interviews in each setting.

### Data processing and analysis

The interviews were transcribed into written text for study. Interviews from the audio-recorder were reviewed several times to obtain verbatim accounts of the discussions. All redundant or overlapping statements were removed, leaving only those points pertinent to the study. These points were later summarised and presented as data for the research. Thematic analysis was employed for data analysis according to Braun & Clarke [12]. These phases included familiarising one’s self with the data, generating initial codes, searching for themes among the codes, reviewing the themes, defining and naming the themes, and producing the final report.

### Robustness and reliability of approach

Validity and reliability have been viewed as concepts through which research methods, data analysis and study findings can be “verified” or “reproduced” and deemed “accurate” [13]. Reliability and validity were achieved by eliminating bias and increasing the researcher’s truthfulness about a proposition related to the social phenomenon, ensuring credibility, trustworthiness, transferability, dependability, and conformability of the research study [14].

### Reflexivity

Reflexivity is defined as a set of continuous, collective, and multifaceted practices through which researchers can consciously critique, appraise, and evaluate themselves about their subjectivity and context that influence the research processes [15].

Olmos-Vega [16] recommended different ways of ensuring reflexivity, some of which were utilised in this study, such as keeping a research diary; recording and transcribing the interviews by two members of the research team; and making sure the process of interpretation was explained explicitly and articulated clearly to the women. In this study, after every interview, the researchers documented their observations and the participants’ reactions in a field diary. Personal feelings about the interviews were reflected on and any bias that may have been unconsciously adopted, noted and eliminated. This helped them to approach subsequent interviews with more reflexivity.

## Results

Table 1 illustrates the socio-demographic characteristics of women who participated in the study to determine the utilization of TMs for the management of AIP. The mean maternal age of the participants was 28.3 years (SD = 6.94), with a 95% confidence interval of 24.97 to 31.66 years, indicating that the women were within their reproductive years. The majority of the women were married 14, (77.78%). In terms of their educational levels, more than half of the women 10 (55.6%) had completed Junior High School, while 3 (16.67%) had no formal education, and nearly a quarter had reached Senior High School (22.22%). Most (94.44%) of the women were not formally employed. They were either fishmongers or were engaged in petty trading. Half (50%) of the women earned below GHS 500 monthly, indicating a low socio-economic status of the women.

**Table 1:** Socio-demographic characteristics of women.

Variable	n (%)	Median (IQR)/ Mean (SD)	95% CI
Maternal age (years)		(28.32, 6.94)	(24.97, 31.66)
Marital status			
Yes	14 (77.78%)		
No	4 (22.22%)		
Educational level			
No formal education	3 (16.67%)		
Primary	1 (5.56%)		
Junior High School	10 (55.56%)		
Senior High School	4 (22.22%)		
Occupation			
No occupation	1 (5.56%)		
Formal	–		
Non-formal	17 (94.44%)		
Monthly Income (GHS)			
Below 100–499	9 (50.00%)		
GHS 500–999	5 (27.78%)		
GHS 1000 - and above	4 (22.22%)		
Educational level of Partner			
No formal education	1 (5.56%)		
Primary	1 (5.56%)		
Junior High School	8 (44.44%)		
Senior High School	7 (38.89%)		
Postgraduate	1 (5.56%)		
Occupation			
Unknown	1 (5.56%)		
Formal	4 (22.22%)		
Non-formal	13 (72.22%)		
Gravidity			
1 (Primigravidity)	2 (11.11%)		
2–4 (Multigravidity)	12 (77.78%)		
5 and above (Grand-multigravidity)	4 (22.22%)		
Parity			
0 (Nulliparity)	2 (11.11%)		
1 (Primiparity)	8 (44.44%)		
2–4 (Multiparity)	6 (33.33%)		
5 and above (grand-mutiparity)	2 (11.11%)		

Regarding their partners, educational levels were slightly higher, with eight (44.44%) partners having completed Junior High School, seven (38.89%) having completed Senior High School, and only one (5.6%) partner holding a postgraduate degree. The women’s partners were primarily engaged in non-formal work, 13 (72.22%), with only 4 (22.22%) employed in the formal sector. Generally, the educational levels and socio-economic statuses of the women and their partners were low.

The majority of the women were multigravida (2–4 previous pregnancies), accounting for 12 (77.78%), while 4 (22.22%) were grand-multigravida (5 or more pregnancies). Almost half, 8 (44.4%) women were primiparous (one previous birth), 6 (33.3%) were multiparous (2–4 births), 2 (11.1%) were grand-multiparous, and only 11.1% had never given birth (nulliparous). The data suggests that most of the women had had experience with pregnancy and childbirth before and may have encountered TMs in the past.

Three main themes were identified for the study, namely: participants’ knowledge of AIP, their knowledge and use of TM, and the role of their support systems.

The first theme covered women’s understanding of the causes of anaemia in pregnancy and their health-seeking behaviours**.**

**1. Knowledge of Anaemia in Pregnancy**

The participants had diverse views about AIP; they coined their own terms to represent the meaning of the condition. Some of the women explained AIP as a condition in which pregnant women had “shortage”, “reduced” or "low blood volume". One of the participants said AIP based on her own previous experience.

**1.1 The Women’s Views about Anaemia in Pregnancy**

*Yes, I know about anaemia. I know it well because in my previous pregnancies, I encountered anaemia. Normally, anaemia means a shortage of blood…. P8*

*When I came here, I was told that my blood volume had reduced. When they say you have anaemia, it means that your blood volume is too low... sic P13*

**1.2 Knowledge of Causes of Anaemia in Pregnancy**

The women were knowledgeable about the causes of AIP. Four leading causes of AIP were identified, namely: poor medication adherence, inability to eat well, stressful work and other medical conditions.

**1.2.1 Medication Non-adherence**

The first important cause of AIP identified by the women was non-adherence to medication given at a health facility. The women claimed that when healthcare professionals prescribed medications, some pregnant women did not adhere to the medication regimen, putting them at risk of becoming anaemic.

*If she [pregnant woman] doesn’t take the medicines that they [healthcare professionals] give to her, her blood volume will reduce. sic P6*

*… then the medications that they [healthcare professionals] will prescribe for you [pregnant woman], if you [pregnant woman] try and purchase and take them[medication], you will do very well. You [pregnant woman] will not become anaemic. If they [healthcare professionals] give it to you [pregnant woman] and you don’t take it, that is what makes you lose blood... You will have low blood. sic P7*

**1.2.2 Inability to eat well**

The second major perceived cause of AIP, according to the pregnant women, was poor eating practices. They stated that they if pregnant women were unable to consume nutritious food, it could result in a reduction in blood volume.

*When you come to the hospital, the doctors will advise you to eat well, but if you don’t eat well, it can lower your blood levels. P3*

*When you have anaemia, it may be because you’re eating pattern is bad, you don’t eatwell. This is what will let someone have anaemia … For some people, they don’t eat foodsthat will give them blood … so their blood volume will reduce. P6.*

**1.2.3. Stressful work**

Few participants claimed that AIP was caused by pregnant women being engaged in stressful work.

*It [AIP] can occur when the woman does stressful, complicated, or hard work P1.*

*It can occur if the pregnant woman does hard or stressful work or doesn’t take her drugs P4*

**1.2.4. Other medical conditions**

Lastly, the women perceived that conditions such as malaria and persistent vomiting could cause anaemia.

*Malaria can also make your blood levels go down. P3*

*We can’t eat well, and the little I eat, too I will vomit it again and that vomiting, vomiting…P8*

The second theme focused on their knowledge of and the utilization of TMs. The subthemes included their knowledge of dietary and non-dietary TMs, preparation of TMs, negative consequences and reasons for non-usage of TMs.

**2. Knowledge and utilization of traditional medicines**

Many pregnant women described various types of TMs they knew and their preparations. Some of these medicines were derived from the whole plants, leaves, tree bark, shrubs, vegetables and herbs. They have been divided into dietary and non-dietary TMs.

**2.1 Traditional Dietary Medicines**

Some pregnant women expressed how they use local plants and vegetables to reduce anaemia, including green leafy vegetables like cassava leaves [*Manihot esculenta*], the leaves of the eggplant [*Solanum macrocarpon*] and the leaves of the groundnut plant [*Arachis hypogaea*]. They believed it made their babies stronger and purified their blood.

*I know about only kontomire [Colocasia esculenta] and the ones used to prepare food. I also know about cassava leaves and bitter leaf [Vernonia amygdalina]. P3*

*When I came to the hospital, I was told to eat “moduru” [Solanum torvum] in addition to the medicine they gave me… That “moduru” is what they said gives blood. You will boil it and add milk to it, but I don’t like it when mixed with milk, so it’s only “moduru” that I used. Then there is okro [Abelmoschus esculentus] and “ayoyo” [Corchorus olitorius] is also good, so I eat that as well. P13*

**2.2 Non-dietary traditional medicines and their uses**

Some women also mentioned several local herbs and non-dietary TMs known to them, such as akokornyidam, moringa, neem tree, aborwombaaguwakyir, mpatuwanswei and their uses. Their uses included several perceived benefits, including relieving fever, abdominal aches and pains, strengthening babies, hastening childbirth, and managing AIP.

*Yes, it is called “Akokornyinida”. It is said that when your pregnancy reaches 7 months, you use it as an enema; it was used by our mothers when they got pregnant. For some women, their babies are not strong, so when they take these herbal medicines, it makes them stronger. P3*

*Yes, the ones I know include moringa and the “neem tree.” They say moringa is used to treat malaria, and the “neem tree” is used to treat fever. With these types of medicines, the treatment depends on the specific disease. Other times, when the disease doesn’t go away after taking hospital medications, you must boil these herbs, and when you drink them, the disease will go away (you will be healed). P4*

**2.3 Preparation of traditional medicines**

Various methods were used for the preparation and use of TMs by these women. These methods include boiling, brewing and filtering, sitz baths, bathing, enema administration, mixing with either milk or tin tomatoes [tomato paste] and grinding to make a paste.

*The other day, someone told me that as a pregnant woman, when you have anaemia, you should boil the leaves used in preparing “sobolo” [Hibiscus sabdariffa] and drink the filtrate. Others include “waakye leaves” [Sorghum bicolor], you boil it and drink it. Other people say you can mix it with milk or tin tomatoes and drink it. It will raise your blood level. P4*

*They made me sit on some of them [sitz bath], she mixed it with hot water, and then I sat on it [sitz bath]. I also drank some and bathed with another. P8*

**2.4 Negative Consequences of Traditional Medicine Use**

While many women perceived that TMs were efficacious in preventing AIP, others also expressed their concerns about the adverse side effects of TMs. They complained about some side effects to the mother, including itching, vomiting, dizziness, weakness, and other potentially serious effects, such as abortion, reduction in the quality of breathing (dyspnea), jaundice and deformities to the newborns.

*My suggestion is that when someone wants to terminate a pregnancy, she will go and take that medicine that they said pregnant women should not take, and when that happens, you are only worrying yourself, P12.*

*Madam, there is no herbal medicine that gives blood. The consequences are a lot; it is just that we ignore them because the effects may not be felt now, but as time goes on, they can make you feel weak. You can’t walk well. I’m saying this because I know someone like that in my house. When she drinks herbal medicine and you talk to her about it, she will say “ohh it’s our forefathers that gave it to us” and now, she can’t walk well. But when you look at her, she’s not very strong. She doesn’t eat well. And for someone like that, it will be difficult to have enough blood. P18*

**2.5 Reasons for not using Traditional Medicine**

One other participant stated that she did not use TMs because of the fear of using medication (herbs) that have been sprayed with weedicides. She shared her personal experience as follows:

*I used it alongside the hospital medicine. But today, due to spraying (weedicides), I have stopped. The doctor has made us aware that they are not helpful. True to that, When I gave birth to my third born, it didn’t help me so I have made up my mind not to use it again. I used it as an enema, but I realised that where I went to pluck the leaves was sprayed (with a weedicide). My baby was not as strong as I desired. In fact, I was admitted to the hospital for about 1 month and two weeks, so it made me suffer. P16*

### Perceptions of mixing Traditional and Antenatal Routine Medicines

Some of the mothers did not take the antenatal routine medication, which led to anaemia in pregnancy. Others combined the TMs and the antenatal medications for profound effects. Some of the women did not mix the medication because of the perceived side effects that may be associated with their combination.

*It helps a lot when you take it with the hospital drugs. But for some people, when they are taking the herbal medicine, they don’t take the hospital medicine again. I took it with the hospital drugs. When I went to the hospital, I was told that my blood volume was low. P12*

*You can only use these medicines when your pregnancy is 1–3 months old. When your pregnancy is 5 months or older, you cannot use these medicines. At this age of the pregnancy, you can only use the hospital medicine. However, we deal with time; hospital medications last for eight hours, so we apply the same principle to herbal medicines. We don’t mix them; there are limits to them, so we do not mix. P15*

The third theme explored the support, which reflected social and contextual factors that either promoted or restricted the use of TM. Subthemes in this area included support from family, healthcare workers, and the community.

**3. Support**

The women stated that they were encouraged by partners, family and friends, and community members to take their TMs during pregnancy.

**3.1 Family and friends’ support**

Family members prepared and encouraged pregnant women to consume and use TMs during pregnancy. Some family members even insisted that the pregnant woman should take the TMs.

*I remember that my auntie did [prepared] some [herbal concoction] for my sister some time ago, which gave her blood, but I don’t know its name. She boiled it and gave it to her to drink, and it would give her blood. And yes, after she took it, they said everything was fine… So she even went to get some for me, which I should boil and drink, or when I want to cook, I should grind it and add it to my soup. However, I didn’t do it. She kept forcing me to take the herbal medication…P15*

They also claimed that the family members offered spiritual support. One participant stated that:

*There is no such medicine in my family, but the family head has some water that, when someone is sick, she will go to him for him to pray for her and give her some of the water. That also helps you to deliver easily. I believe in it; I have used it before. Sometimes, we put our faith in it. P18*

**3.2 Health workers**

Some of the women stated that the health workers checked their anaemic status, encouraged them, educated them about their medications, and advised against using TMs. They were also instructed to eat nutritious foods, sleep in Long Lasting Insecticide Treated Nets (LLITNS), and rest to reduce AIP.

*The health workers also encourage me to take my drugs and to eat well so that my blood levels will go up P1.*

*For the current pregnancy, I was told not to use herbal medicine when I came for the antenatal clinic, so I haven’t used any herbal medicine. P3*

**3.3 Community support**

The community offered support to pregnant women by organizing communal labour to eliminate mosquitoes and education of anaemia in pregnancy at the information centre

*They organize communal labour and weeded our surroundings to keep us from mosquitoes. P3*

*No, the one I have heard about they said pregnant women do not take it. I heard it over the information centre. P12*

### Traditional medicine integration

When asked about TM integration, the women were more concerned with the suitability with the TM to the pregnant woman, and its compatibility with other orthodox medicines.

*When the government wants to do that, they should check whether the person’s system is suitable for that herbal medicine before they give it to you. So, it is good to that the integration is done. P4*

*When you combine the two, it won’t be okay. You’re taking hospital medications and at the same time taking herbal medications. Unless the doctors give me advice, I don’t see how that can be done. So, unless the doctors advise us, I’m not a doctor, and I don’t know about that. P5*

## Discussion

### Knowledge of anaemia in pregnancy

Most participants described anaemia as “shortage” or “reduced blood”. Findings from another African settings stated that anaemia was conceptualised as “lack of blood” [17]. While semantically simple, this explanation reflects embodied knowledge grounded in women’s lived experiences of weakness and prior pegnancies. Such understandings can be leveraged in clinical education, as aligning biomedical explanations of haemoglobin with women’s metaphors may enhance adherence to preventive measures [18].

### Perceived causes of anaemia

Four major causes were identified: non-adherence to prescribed medication, poor diet, stressful work, and other conditions such as malaria and vomiting. Many women emphasised medication non-adherence. This perception aligns with evidence that poor compliance with iron-folic acid supplementation significantly contributes to anaemia in pregnancy in Ghana [19]. Dietary insufficiency was also repeatedly cited. Poor dietary diversity has been linked to high anaemia prevalence in West Africa [20], confirming the salience of this explanatory model. Interestingly, participants also attributed anaemia to “stressful work”, reflecting socio-cultural interpretations of vulnerability in pregnancy. While not directly supported by biomedical literature, the interaction of physical exertion, nutrition, and infection influences maternal health outcomes. [21]. The identification of malaria as a contributor reflects biomedical consensus, as malaria-related anaemia remains a major challenge in Ghana despite preventive measures such as intermittent preventive treatment for malaria [22].

### Knowledge of and practical uses of traditional medicine

Participants demonstrated detailed knowledge of both dietary and non-dietary TMs. Dietary practices included the use of green leafy vegetables (e.g., cassava leaves, kontomire, bitter leaf) and culturally specific foods such as *Solanum torvum* (“moduru”), believed to “give blood” and “make babies stronger”. While some of these foods are rich in micronutrients, the biomedical evidence is mixed; for example*, S. torvum* has shown potential to improve haemoglobin levels in adolescent populations [23] but safety and efficacy in pregnancy remain under-researched.

Non-dietary remedies included herbs such as moringa and neem, used for conditions like fever and malaria [24]. However, women were also aware of adverse consequences, citing side effects ranging from dizziness and weakness to abortion and neonatal deformities [25]. These accounts mirror research highlighting both the widespread use and potential risks of herbal medicines in pregnancy, including herb–drug interactions and uterotonic effects [26,27]. The ambivalence expressed underscores the tension between cultural knowledge and biomedical caution, a key theme in pluralistic health systems [28].

### Relational and systemic influences on care-seeking

Interactions among family, community, and the healthcare system shaped women’s practices. Families often prepared herbal remedies, reflecting the centrality of kinship in health decision-making [29]. Community-level support, such as environmental sanitation to reduce mosquitoes, was also reported, aligning with collective health promotion strategies documented in rural Ghana [30].

During antenatal visits, healthcare professionals cautioned pregnant women against certain traditional medicines due to their potential effects on the fetus [31]. The pregnant women were encouraged to eat nutritious foods and use LLITNs to prevent malaria and anaemia. This mix of endorsement, negotiation, and caution from healthcare workers added another layer of influence on women’s decisions about using traditional medicine.

### Integration of traditional and orthodox medicines

Participants expressed a range of opinions on blending traditional and biomedical treatments. Some discussed safe time-separation strategies, whereas others opposed concurrent use due to possible risks. A small number were open to integration, provided they were supervised by biomedical experts, reflecting ongoing debates in Ghana about formally incorporating TM into health systems. While integration could improve safety and monitoring, worries about pharmacological interactions remain. For example, tannins and polyphenols in many herbal remedies can interfere with iron absorption, possibly decreasing the effectiveness of supplements [32]. Ghana has integrated TMs into the formal health system by developing policies and regulatory structures, strengthening standardisation, and embedding herbal services within primary healthcare. This integration has been reinforced by training medical herbalists through recognised institutions and enabling them to work alongside other healthcare professionals in clinical settings. Even so, there is still limited evidence on how pregnant women use these integrated services and how satisfied they are with the care they receive.

### Review of traditional medicines mentioned in the study

The reviewed TMs are highly regarded in ethnopharmacology for managing anaemia during pregnancy, mainly due to their haematinic, hepatoprotective, and micronutrient-enhancing properties. Nutrient-rich vegetables such as *Corchorus olitorius* (ayoyo) and *Colocasia esculenta* (kontomire) contain high levels of iron, folate, and vitamins A and C, which are crucial for erythropoiesis and antioxidant protection when incorporated into the local diet [33–38]. Similarly, medicinal plants like *Moringa oleifera, Abelmoschus esculentus, Hibiscus sabdariffa,* and *Sorghum bicolor* support red blood cell formation and enhance iron absorption, making them valuable dietary components against iron-deficiency anaemia [39–42]. Other plants, such as *Phyllanthus fraternus, Newbouldia laevis, and Solanum species (S. torvum, S. macrocarpon*), are traditionally employed to assist postpartum recovery and promote blood health [33].

Even though their therapeutic benefits are recognised, potential toxicities require careful use. Excessive consumption of *Vernonia amygdalina* may suppress erythropoiesis or cause cardiotoxic and myelotoxic effects [33,43].

Unprocessed *Manihot esculenta* leaves pose a risk of cyanide toxicity [44]. *Azadirachta indica oil* ingestion can lead to severe neurological symptoms [44]. *Solanum macrocarpon* contains glycoalkaloids that may cause gastrointestinal discomfort, and Arachis hypogaea leaves may trigger allergic reactions in sensitive individuals [45]. Proper culinary methods including boiling, steaming, or decoction, may reduce adverse effects and improve TMs [41,46]. Overall, these TMs are culturally rooted, accessible, and nutritionally valuable options that can complement conventional iron–folate therapy in maternal care when used within safe dose limits [47] See Table 2.

**Table 2:** Preferred Traditional Medicines, their Botanical Names, their Anaemia-reducing Properties, their Uses/ Preparation, Toxicity and Adverse Effects.

Name of traditional medicine by Participants	Botanical or scientific name	Anaemia-reducing properties	Uses/Preparation	Toxicity/Adverse effects
Abor womba agu wakyir	*Phyllanthus fraternus*	Has been shown to have hepatoprotective and hematinic effects [48]	Herbal infusion or powder [49]. Used in herbal infusions for anaemia and hepatitis [50]	No substantial adverse effects [46]
Akokornyidem	*Newbouldia laevis*	Used for fertility and menstrual regulation; may help red blood cell recovery [51]	Infused in tea, decoctions for fertility and uterine health [52]	Generally low [53], however, it has hepatotoxic and biliary toxicity [52].
Ayoyo leaves	*Corchorus olitorius*	Contains vitamins A and C, and iron supports hematopoiesis [33,34]	Leaves are washed in distilled water, sundried, milled or blended in soup [36]. Leaf extract may be a potential source of antioxidants, anti-inflammatories, anticancer and good for the cardiovascular system [54]. Safe and potentially postprandial antidiabetic effects [55]. Used in soups to support blood formation [33]. Leaf extracts may be a potential source of anti-inflammatory, anticancer, and cardiovascular benefits.	Low; considered safe when cooked [55].
Bitter leaves	*Vernonia amygdalina*	May inhibit erythropoiesis; however, it has hepatoprotective and anti-anaemic properties in controlled doses [33,56].	Decoction, cooking, infusion [57]. For the treatment of Malaria [58], intestinal worms [] [59], antioxidants [53], and to increase the nutritional value of food.	Generally low; however, excessive consumption may suppress erythropoiesis due to low iron content Click or tap here to enter text. [33]. High doses might inhibit erythropoietin production, potentially leading to anaemia, especially in menstruating and pregnant women. Also known to cause myelotoxicity, cardiotoxicity in severe cases [43].
Cassava leaves	*Manihot esculenta*	Contains calcium and iron [60], which prevent anaemia and bone-related conditions [61].	Serves as nutritional supplementation and treatment of malnutrition [62]. Stewed, soaking and boiling [63]	Cassava leaves exhibit phytotoxicity, and their use is limited by cyanide toxicity [64]. Consumption of raw or improperly processed cassava can lead to cyanide poisoning, causing symptoms like vomiting, diarrhoea, confusion, dizziness, and loss of consciousness and in severe cases, neurological disorders [65].
Eggplant leaves	*Solanum macrocarpon*	High in iron and antioxidants; may support haemoglobin production [66]. Recommended for anaemic patients and for pregnant and postnatal mothers [67]. Anticancer because of its high composition of phytochemicals. Used in treating nephrotoxicity [45].	Leaf decoction is used in stews or as an ingredient in herbal teas. Stewed or decocted [45].	Low to moderate; contains glycoalkaloids. High intake may cause gastrointestinal disturbances and, in rare cases, toxicity symptoms like vomiting and diarrhoea. [45].
Groundnut plant leaves	*Arachis hypogaea*	Iron-rich foliage may help prevent iron-deficiency anaemia [68].	Extracts used in TMs to enhance sleep [69,70]. Boiled and eaten or dried and crushed.	Low for most individuals; high for those with peanut allergies. Allergic reactions can range from mild skin rashes to severe anaphylaxis [71]
Kontomire	*Colocasia esculenta*	A good source of iron and folate, supporting blood formation and fetal development [72]. Has several pharmacological activities, including antidiabetic/hypoglycaemic, Antimicrobial, antioxidant, Antifungal, anti-inflammatory, Nervine tonic, Hepatoprotective/Antihepatotoxic, and Anticancer properties [37].	Stewed and used as an iron-rich local delicacy [73]. Boiled eaten, pureed or dried into powder [38]	Side effects are not substantial [46]. High doses might inhibit erythropoietin production, potentially leading to anaemia, especially in menstruating and pregnant women [33]. Also known to cause myelotoxicity, cardiotoxicity, and (in severe cases) vasospastic effects [43]. Consumption of raw or improperly processed cassava can lead to cyanide poisoning, causing symptoms like vomiting, diarrhoea, confusion, dizziness, and loss of consciousness and in severe cases, neurological problems. No significant adverse effects reported [74]. High doses may lead to liver and kidney function alterations, and potential miscarriage in pregnant women [75,76]. No significant adverse effects reported [47]. No significant adverse effects reported [41]. May cause mild gastrointestinal discomfort in some individuals. Toxicity is low Moderate if raw; contains calcium oxalate crystals [77].
Moduru/ Bedro	*Solanum torvum*	High iron and folate content; used in traditional postnatal care [78]	Cooked or boiled in palm-nut soup and stews or herbal mixtures [74]	Low; traditionally used in various dishes. No significant adverse effects reported [74].
Moringa leaves	*Moringa oleifera*	Contains iron, calcium, vitamins A & C; effective against anaemia [39]	Tea, soup, or powdered leaf formulation [40]	Generally low; root and bark extracts can be toxic. High doses may lead to liver and kidney function alterations, and potential miscarriage in pregnant women [] [75,76]
Mpatuwanswei	*Alternanthera pungens*	Antipyretic and antispasmodic use [47]. Antimicrobial and antioxidant. Not directly linked to anaemia in pregnancy [79]	Boiled and taken orally for anaemia. Herbal tea or soup	Generally low; limited data available. No significant adverse effects reported [47]
Neem tree leaves	*Azadirachta indica*	Traditionally used for malaria and fever; immunomodulatory and hepatoprotective [80]	Decoction or powder in tea. Bitter tea, steam inhalation, or infusion [81].	Ingestion of neem oil can cause vomiting, seizures, and, in severe cases, toxic encephalopathy [44].
Okro	*Abelmoschus esculentus*	Shown to increase the number of erythrocytes and haemoglobin (Hb) levels in the blood [41]	Consumed as soup or stew, especially in pregnancy [82]	Low; widely consumed as food. No significant adverse effects reported.[41].
Sobolo, Zobo	*Hibiscus sabdariffa*	High vitamin C content aids iron absorption; antioxidant effect [68]	Brewed and infused as a herbal drink and tea made from dried petals [83]	Low; generally safe. However, high doses used for a relatively long term might harm reproductive systems, kidneys, and liver functions [83]
Waakye leaves	*Sorghum bicolor*	Anthocyanins and iron content may contribute to RBC synthesis [42]	Boiled and used as a soup additive or tea.	Toxicity is low [73,84] it contains cyanogenic glycosides.

### Implications for policy and practice

The findings emphasise the importance of developing practice and policy strategies that recognise the widespread use and cultural value of TMs in maternal care, while prioritising safety and efficacy. Midwives and healthcare professionals should implement culturally sensitive counselling methods that respect women’s beliefs and practices, encouraging open dialogue about TM use and providing balanced information on its benefits and risks. Including traditional health knowledge in antenatal education can build trust, facilitate disclosure, and prevent harmful interactions between TM and prescribed medications. At the policy level, integrating TM into formal health systems requires clear regulatory frameworks, standardized preparation procedures, and strong pharmacovigilance systems to ensure safety and quality. Moreover, training healthcare workers in ethnopharmacology and promoting respectful maternity care will help deliver culturally responsive services. These combined measures can unify traditional and biomedical approaches, improve adherence to anaemia prevention strategies, and support holistic maternal health care.

### Strengths and limitations

First, like most qualitative research, the findings are based on the subjective accounts of participants and thus cannot be generalised to all pregnant women in Ghana. Secondly, some participants might have experienced recall bias or social desirability bias, especially when discussing their use of TMs or interactions with healthcare providers. The cultural sensitivity of the subject may have also influenced how openly participants shared their experiences, possibly limiting the depth of some responses.

Despite these limitations, the study has several strengths. It offers an in-depth, contextually grounded understanding of women’s knowledge, perceptions, and motivations regarding the use of TM during pregnancy. The study offers insights that quantitative methods may overlook. The semi-structured interviews created an ambient environment for participants to share cultural beliefs and practices. Additionally, involving different participants from various communities increased the credibility and transferability of the results. Overall, the study provides valuable evidence to inform culturally sensitive maternal health education, improve provider–patient communication, and guide future research and policy development on the safe integration of TM into maternal healthcare in Ghana.

## Conclusion

This study explored the utilization of TMs by women during pregnancy for the management of anaemia in pregnancy, which is deeply rooted in their knowledge, beliefs, and support systems that influence their health behaviours. The results show that perceptions and knowledge of anaemia in pregnancy, issues like poor nutrition, stress, and medication non-adherence, as well as the impact of family, healthcare workers, and community support, are all crucial in guiding maternal health choices. These findings highlight the need to strengthen supervised TMs in biomedical health systems to ensure safe and effective care for pregnant women. Merging respect for local TM practices with evidence-based maternal health. Traditional medicine care is essential. Interventions could promote better health outcomes for mothers and their babies.
